# Pre-clinical medical students’ perception of surgical professions and balance with personal life – a single-centre exploratory analysis

**DOI:** 10.1186/s12909-026-10822-6

**Published:** 2026-11-13

**Authors:** Hannes Becker, Hanna Gött, Jonas Tellermann, Patrick Haas, Constantin Roder, Marcos Tatagiba, Felix Behling

**Affiliations:** 1 Department of Neurosurgery, University Hospital Tuebingen, Eberhard Karls University Tuebingen, Tuebingen, Germany; 2 Department of Neurology & Interdisciplinary Neuro-Oncology, Hertie Institute for Clinical Brain Research, Center for Neuro-Oncology, Comprehensive Cancer Center, University Hospital Tuebingen, Eberhard Karls University Tuebingen, Tuebingen, Germany; 3 Center for Neuro-Oncology, Comprehensive Cancer Center Tuebingen-Stuttgart, University Hospital Tuebingen, Eberhard Karls University Tuebingen, Tuebingen, Germany

**Keywords:** Surgical career, Pre-clinical medical students, Medical education, Gender disparity, Work-life balance

## Abstract

**Background:**

Surgical professions deal with serious challenges to inspire students to pursue a surgical career and counteract demographic change. Students’ interest in sub-specialization is influenced by socio-economic factors including work-life balance, flexible working hours and appreciation. However, robust data about the current perception of surgical professions in the early stage of medical education is lacking so far.

**Methods:**

We performed a voluntary online survey among students who participated in an elective neurosurgical course during their preclinical stage of medical studies. The investigator-developed survey addressed perception of unspecified surgical professions, including personal requirements, profession impact on health and lifestyle and the topic of gender disparities and family planning and was conducted before the students entered their elective course. We also assessed viewpoints on support programs facilitating compatibility of surgical training and family life.

**Results:**

A total of 109 students participated in the voluntary survey (66.1% female, 33.0% male, 0.9% not specified). Overall, 56.0% of respondents reported prior experience with surgical professions and 33.9% planned to pursue a surgical career. Most respondents (68.8%) rated surgical professions as more physically demanding than other specialties, and about half (49.5%) considered them more harmful to physicians’ health. In contrast, only 26.6% rated the mental burden as higher in surgical than in other specialties. In the exploratory gender comparison, female participants more often agreed that male residents have better career chances in surgery, whereas male participants more often responded neutrally to this statement.

Overall, 89.0% of participants expressed interest in unspecified support programs to improve the compatibility of professional and personal life, and 65.1% would be willing to accept a one-year extension of their surgical residency.

**Conclusions:**

In summary, pre-clinical medical students of a preselected cohort tend to rate surgical disciplines as demanding, competitive and difficult to balance with private life. Most participants expressed interest in support programs for combining surgical training and family life which needs further evaluation in future studies.

**Supplementary Information:**

The online version contains supplementary material available at https://doi.org/10.1186/s12909-026-10822-6.

## Introduction

Health care systems in Western Europe have to deal with serious difficulties balancing between higher demand and a shortage of available specialists and resources. Due to the demographic changes towards an ageing population, medical services, including both conservative and surgical procedures, are more often required while socio-economic predictions assume a rising demand during the following decades [1, 2]. However, available and talented future residents who will fill the gaps of an equally aging populations of practicing physicians in all medical subspecialties are rare and are highly sought after [3, 4]. Even more importantly, the current generation’s expectations towards the medical profession have changed significantly over the last few years and strongly influences their choice of sub-specialization [5, 6]. Medical students’ socio-economic considerations include flexible working hours, personal appreciation, as well as a work-life balance including time and capabilities for family planning [7]. Surgical training programs, especially in highly specialized surgical subspecialities as neurosurgery, are distinguished by especially long working hours, hierarchical and socially challenging mentor relationships, and high personal commitment combined with unpredictable working schedules [3, 7, 8].

Therefore, surgical subspecialties are becoming increasingly unattractive among medical students and surgical departments are facing difficulties acquiring motivated and talented residents to pursue a surgical career. Even though neurosurgery is traditionally known as a highly competitive subject, especially in Anglo-American countries, recent statistics show, that fewer students favor a career in neurosurgery in Western-European, as well as in high- and middle-income countries of different cultural backgrounds [9, 10].

Students’ perceptions of surgical disciplines are predominantly influenced by prejudicial attitudes and limited negative personal experiences during their medical training [11]. As neurosurgery in many places is not a mandatory course during medical school, exposure of medical students towards the neurosurgical working environment is sparse and their perception of the subjects’ working conditions as well as the role of a neurosurgeon is often impacted by a combination of observed or empirically supported working conditions, personal experiences, media portrayals, and persistent stereotypes [9].

Therefore, we aimed to assess students’ perceptions of surgical sub-specialties at an early stage of medical education to delineate potential barriers limiting their surgical interests, broad gender-based differences and illuminate possible solutions for improving both the surgical role model perception and its implementation in surgical education.

## Materials and methods

A web-based survey was constructed using the online platform SurveyMonkey (SurveyMonkey Inc., San Mateo, California, USA). The contents of the survey were based on our experiences gained during a specific preclinical elective course for medical students. For many years we have offered an elective course where medical students shadow our neurosurgical on-call team for 12 h and afterwards reflect on perceived surgical stereotypes, physician roles, interdisciplinary communication and their general interest in the surgical field (reflection questions are shown in Supplementary Material 1). We were surprised by the negative stereotypes and conceptions about the daily work in neurosurgery and in general surgical subspecialists and constructed our questions based on these expressions. The survey is an investigator-developed questionnaire focusing on a structured evaluation of students’ interest in the field of surgery, their perception of requirements and challenges when pursuing a surgical career and their interest in entering a surgical training program. It was distributed to medical students who participated in the mentioned elective course between October 2023 and February 2025 (*n* = 130). Participation was completely voluntary and anonymous. If the students decided to participate, the survey was administered and had to be completed before the 12-hour shadowing in the neurosurgical department, so that the survey results would not be confounded by impressions gained during the elective. We provide the respective invitation e-mail as well as our questions of the survey within a supplementary file (Supplementary Material 2). No personal data were collected. We constructed 16 out of 18 questions using a five-tiered Likert scale for either expressing agreement or disagreement on different statements or interests. Two of the 18 questions were dichotomous, asking for agreement or disagreement. We provide the complete survey and raw data as a supplementary file (Supplementary Material 2–3). Overall, 109 students participated in the survey (response rate 83.8%). Study items were analyzed for the complete cohort and, as an exploratory analysis, stratified by gender (question 1). One student who did not report their gender was excluded from this subgroup analysis.

Exploratory comparisons between female and male participants were performed using Pearson’s χ² test and Fisher’s exact test on the full distribution of the five Likert categories. In addition, odds ratios (ORs) were calculated for agreement versus disagreement. For this purpose, agreement was defined as the Likert categories “strongly agree” and “agree”, and disagreement as “disagree” and “strongly disagree”. Neutral responses were not included in the OR calculations in order to provide a straightforward dichotomized analysis as part of this exploratory approach. Consequently, each OR is based only on those female and male participants who either agreed or disagreed with the respective statement, so that the number of participants contributing to each OR differs between items. ORs are presented as the odds of agreement among male compared with female participants, together with 95% confidence intervals (CIs), and the Wald test was used to assess their statistical significance. We considered p values < 0.05 statistically significant. No adjustment for multiple testing was performed; given the number of comparisons, all p values should therefore be interpreted as exploratory. Detailed results of all gender comparisons are provided in Supplementary Material 4.

The anonymous and completely voluntary survey was performed in accordance with the Declaration of Helsinki. The local ethics committee reviewed the project and, as the survey was anonymous and voluntary, waived formal consent and considered full ethics approval unnecessary (ethical committee Tuebingen/ 242/2024B02).

**Fig. 1:**
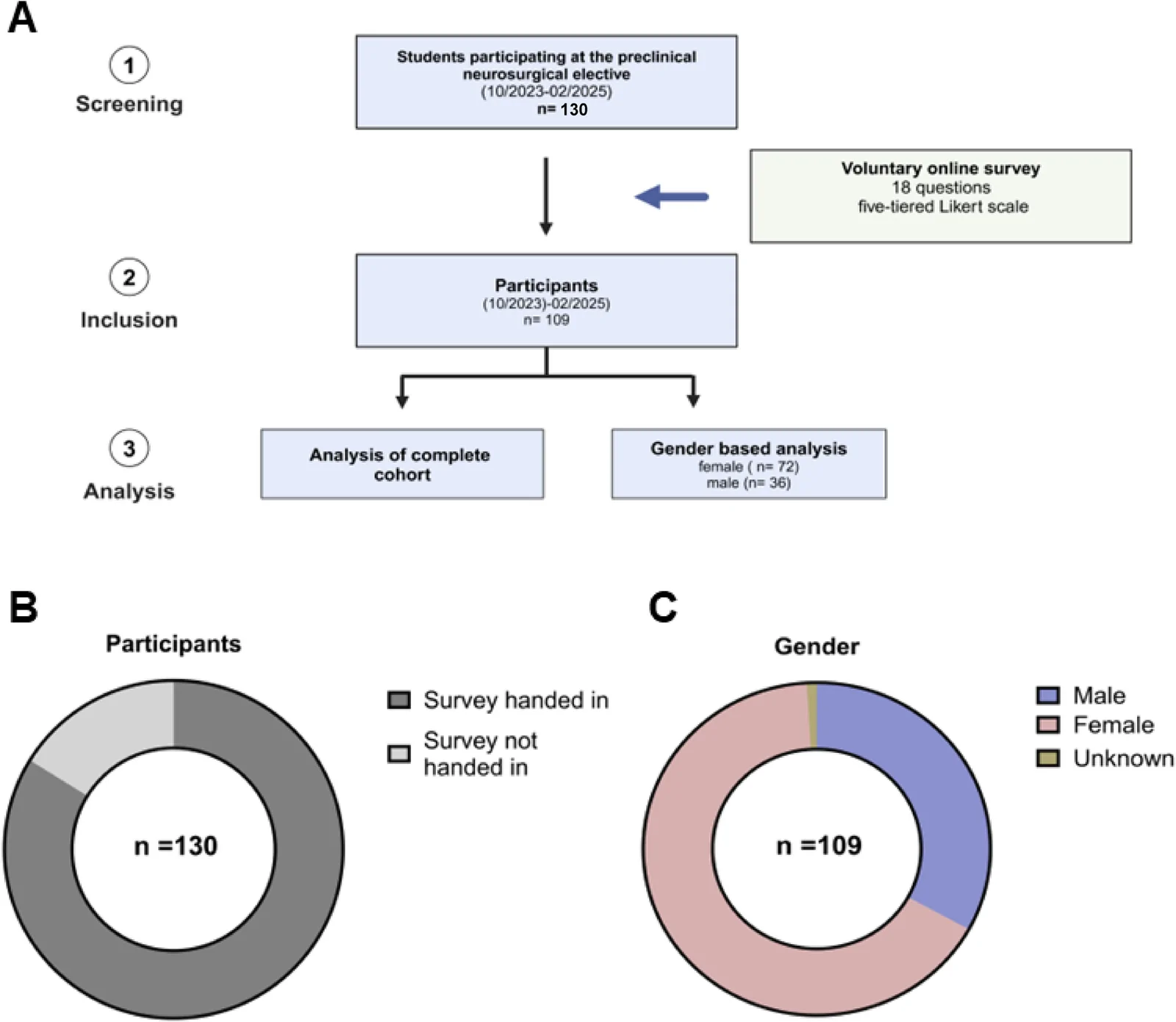
**A** Flow chart depicting conceived study design with **B** overall participation in online survey and **C** gender distribution of participants

Data processing was done with GraphPad Prism 10 (GraphPad Software, San Diego, CA, USA) and Microsoft^®^ Excel (Version 16.93.1, Microsoft, Redmond, Washington, US). Figure 1A was created using Biorender.com.

## Results

### Descriptive data of respondents and surgical interest

**Fig. 2:**
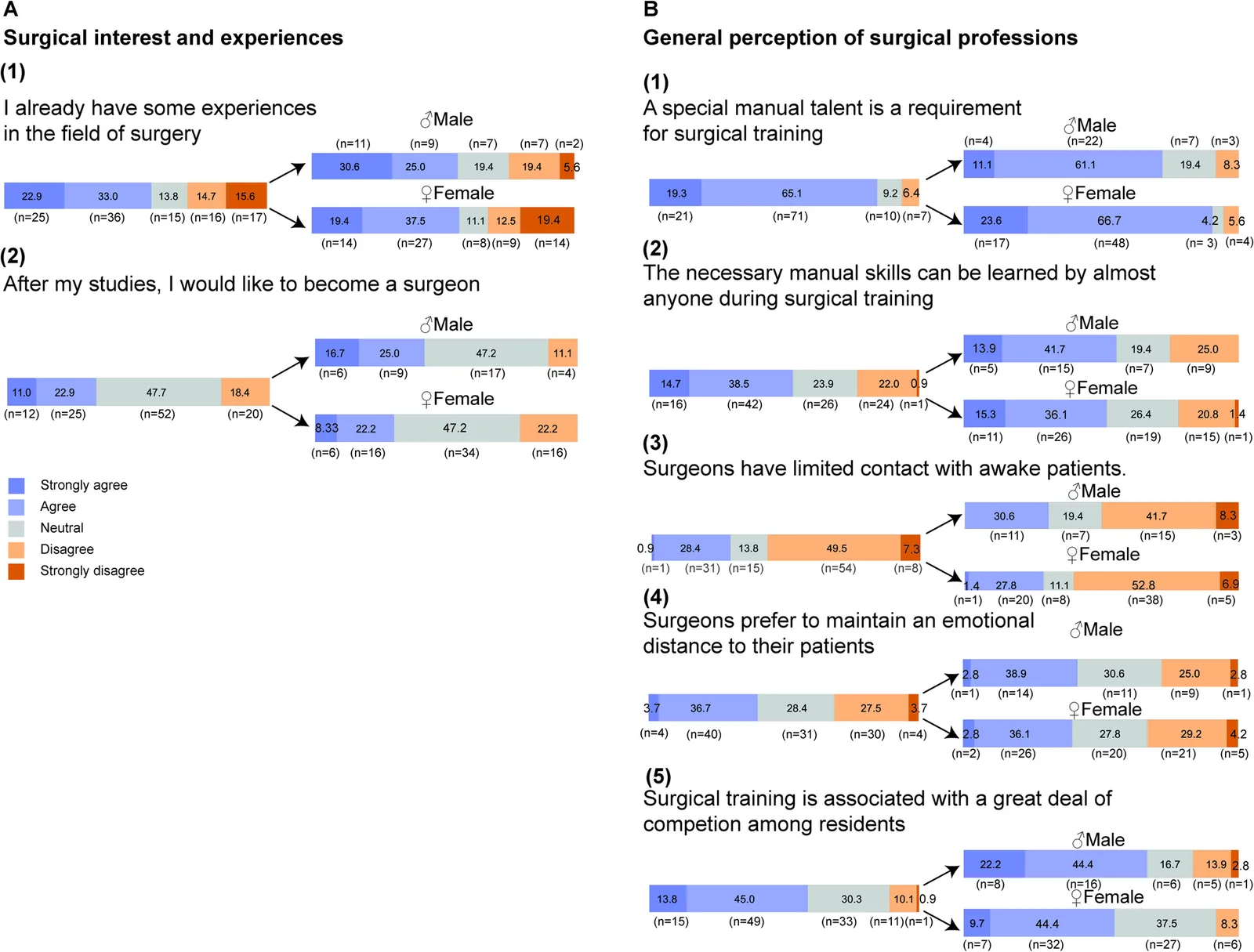
Stacked bar charts of students’ answers in the field of **A** Surgical interest and experiences and **B** general perceptions of surgical professions. Answers are visualized in male and female subgroup accordingly. Grade of agreement as color-coded underneath. Absolute numbers as well as percentages are written in the respective diagram accordingly. Single statements are labelled with numbers in brackets

Overall, 130 students in their preclinical stage of medical studies took part in the elective neurosurgical course between October 2023 and February 2025 (Fig. 1A). Of these, 109 students (83.8%) completed the voluntary online survey (Fig. 1B). All submitted surveys were complete and were included in the analysis. Seventy-two participants were female (66.1%), 36 were male (33.0%), and one student did not specify their gender (Fig. 1C). Overall, 56.0% of participants reported prior experience in surgery, and 33.9% aspired to a career in surgery (Fig. 2A). In the exploratory gender-stratified analysis, this proportion was numerically higher among male than among female participants (41.7% vs. 30.6%), but the difference was not statistically significant (*p* = 0.371, Fisher’s exact test).

### General perception of surgical professions

Next, students were asked about their perceptions of the manual requirements of surgery and of surgical character traits (Fig. 2B). Most respondents (84.4%) agreed that a special manual talent is required for surgical training (Fig. 2B [1]). At the same time, 53.2% agreed that the necessary manual skills can be acquired by almost anyone during residency, whereas 22.9% disagreed (Fig. 2B [2]). More than half of the respondents (56.9%) disagreed with the statement “surgeons have limited contact with awake patients”, whereas 29.4% agreed. A larger proportion agreed that surgeons prefer to maintain an emotional distance from their patients (40.4%) than disagreed (31.2%). Most participants (58.7%) assumed strong competition among surgical residents, and only 12 students (11.0%) disagreed. Male students more often strongly agreed with this statement (22.2% vs. 9.7%), whereas female students more often responded neutrally (37.5% vs. 16.7%). This difference was only borderline significant (*p* = 0.049, Fisher’s exact test) and, given the number of unadjusted comparisons, should not be overinterpreted (Supplementary Material 4).

### Surgical profession impact on health and lifestyle

**Fig. 3:**
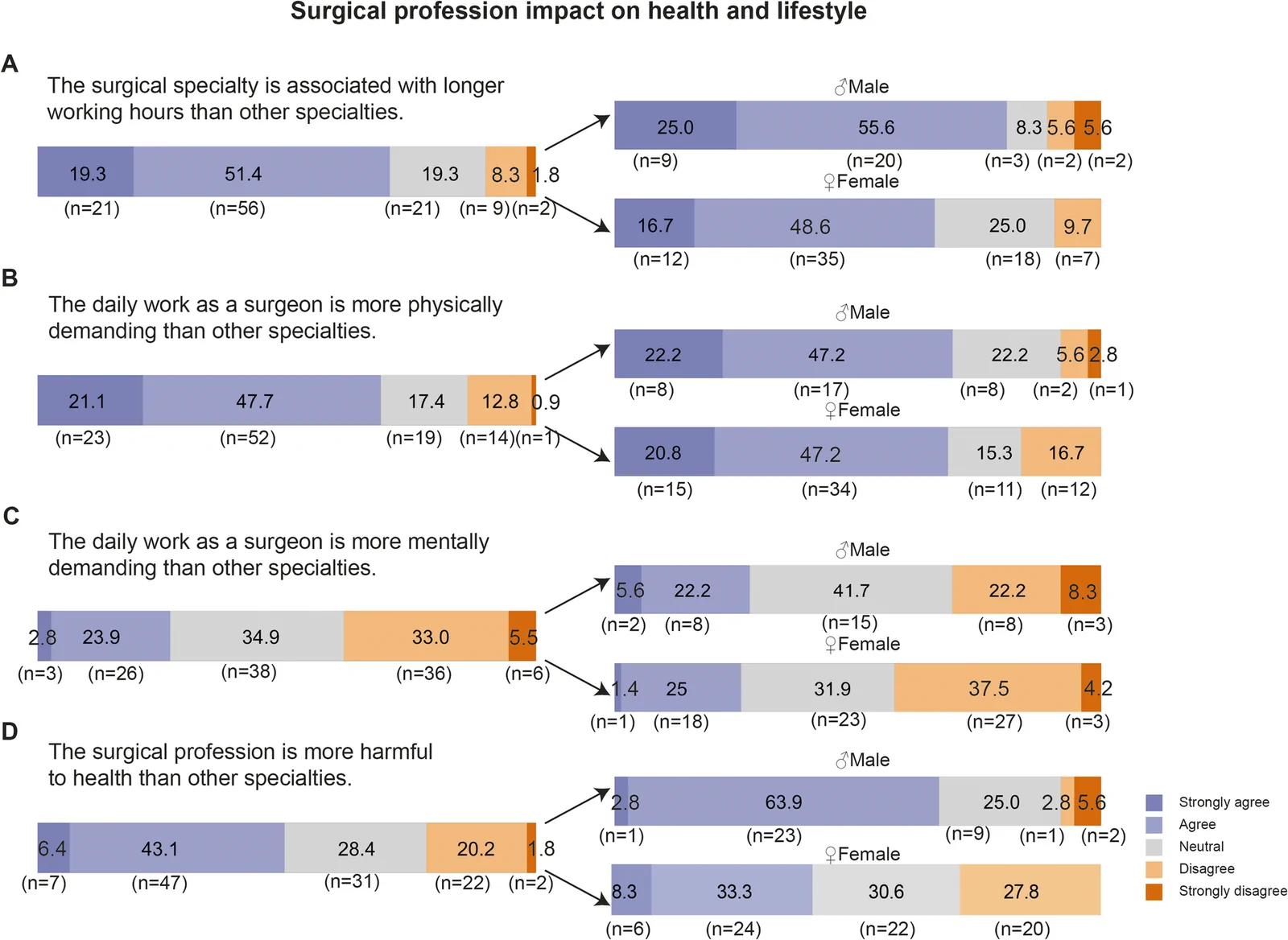
Surgical profession and impact on health and lifestyle. Stack diagrams of whole student population (left) and subgroups female and male (right) are shown. Grade of agreement in regard of (**A**) working hours, (**B**) physical demand, (**C**) mental demand and (**D**) consequences on surgeons’ health in surgical professions is shown. Grade of agreement as color-coded underneath. Absolute numbers as well as percentages are written in the respective diagram accordingly

When asked about the impact of entering surgical training on health and lifestyle, 70.6% of students associated a surgical sub-specialization with longer working hours than other medical professions (Fig. 3A). Regarding physical and mental demands, 68.8% of students rated surgical professions as more physically demanding than other specialties (Fig. 3B). In contrast, only 29 students (26.6%) rated the mental burden of daily work as higher in surgical than in other specialties, while 38 students (34.9%) remained neutral and 42 (38.5%) disagreed (Fig. 3C). Overall, 49.5% (*n* = 54) of respondents rated the surgical profession as more harmful to the health of practitioners than other medical fields (Fig. 3D). Responses to this statement differed between male and female participants (*p* < 0.001, Fisher’s exact test): 66.7% of male students agreed and 8.3% disagreed, whereas 41.7% of female students agreed and 27.8% disagreed. Accordingly, male students had higher odds of agreement than female students (OR = 5.33, 95% CI 1.44–19.75, *p* = 0.013); however, the wide confidence interval indicates considerable imprecision of this estimate.

### Gender disparities and family planning

**Fig. 4:**
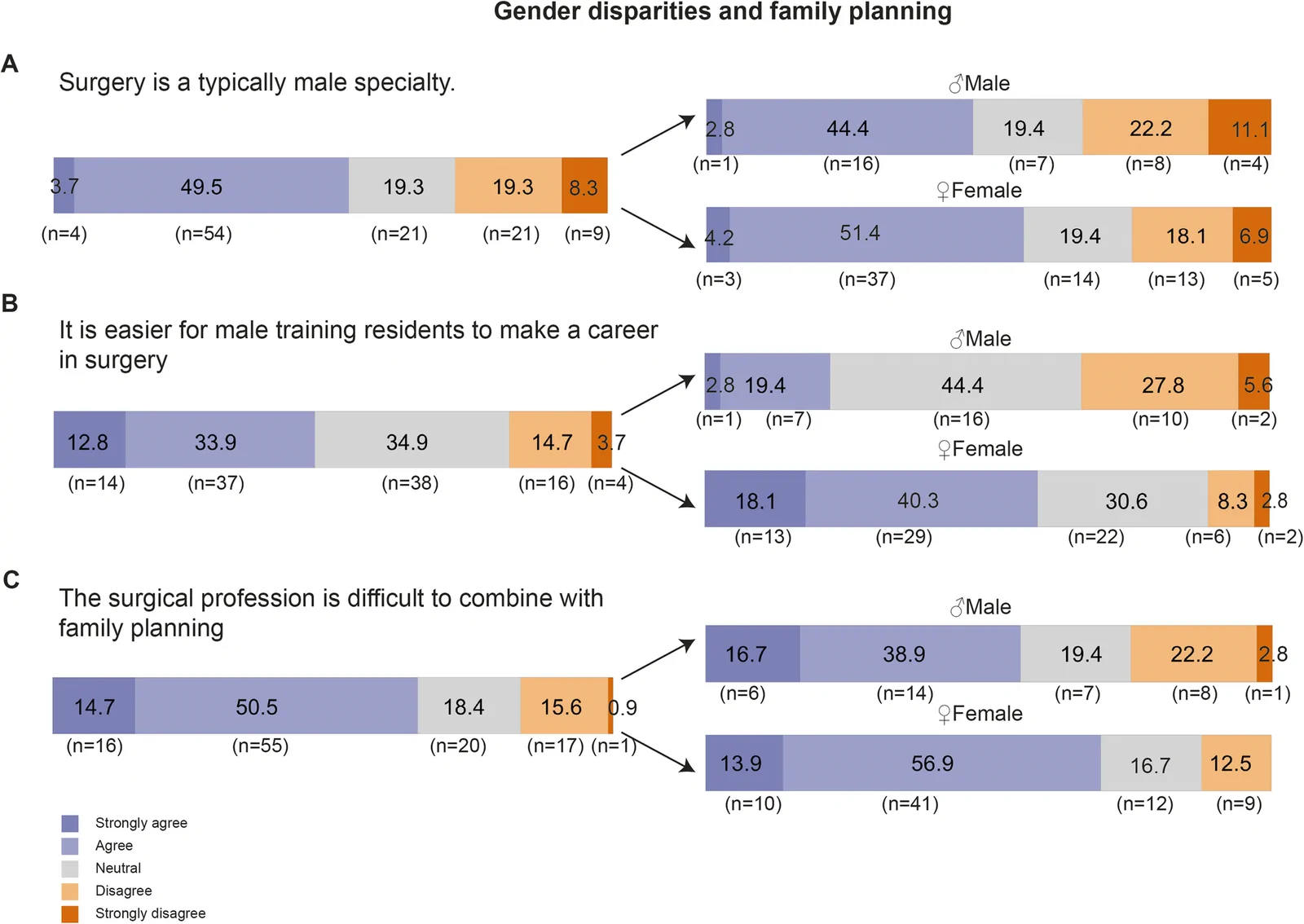
Gender disparities and family planning. Stack diagrams of whole student population (left) and subgroups female and male (right) are shown. Grade of agreement in regard of surgical subspecialities as (**A**) men dominated (**B**) easier career development for male residents and (**C**) difficult to combine with family planning is shown. Grade of agreement as color-coded underneath. Absolute numbers as well as percentages are written in the respective diagram accordingly

Next, participants were asked about their perceptions of gender disparities and family planning in surgical professions (Fig. 4). More than half of the respondents (53.2%) considered surgery a typically male specialty, whereas 27.5% disagreed (Fig. 4A). Responses to the statement “male residents have an advantage in pursuing a career in surgery” differed between male and female students (*p* = 0.002, Fisher’s exact test): 58.3% of female students agreed, compared with 22.2% of male students, 44.4% of whom remained neutral (Fig. 4B). Male students had lower odds of agreement than female students (OR = 0.13, 95% CI 0.04–0.43, *p* = 0.001). Overall, 65.1% of students considered the surgical profession difficult to combine with family planning (Fig. 4C). Agreement was numerically higher among female (70.8%) than among male students (55.6%), but this difference was not statistically significant (OR = 0.39, 95% CI 0.14–1.09, *p* = 0.073).

**Fig. 5:**
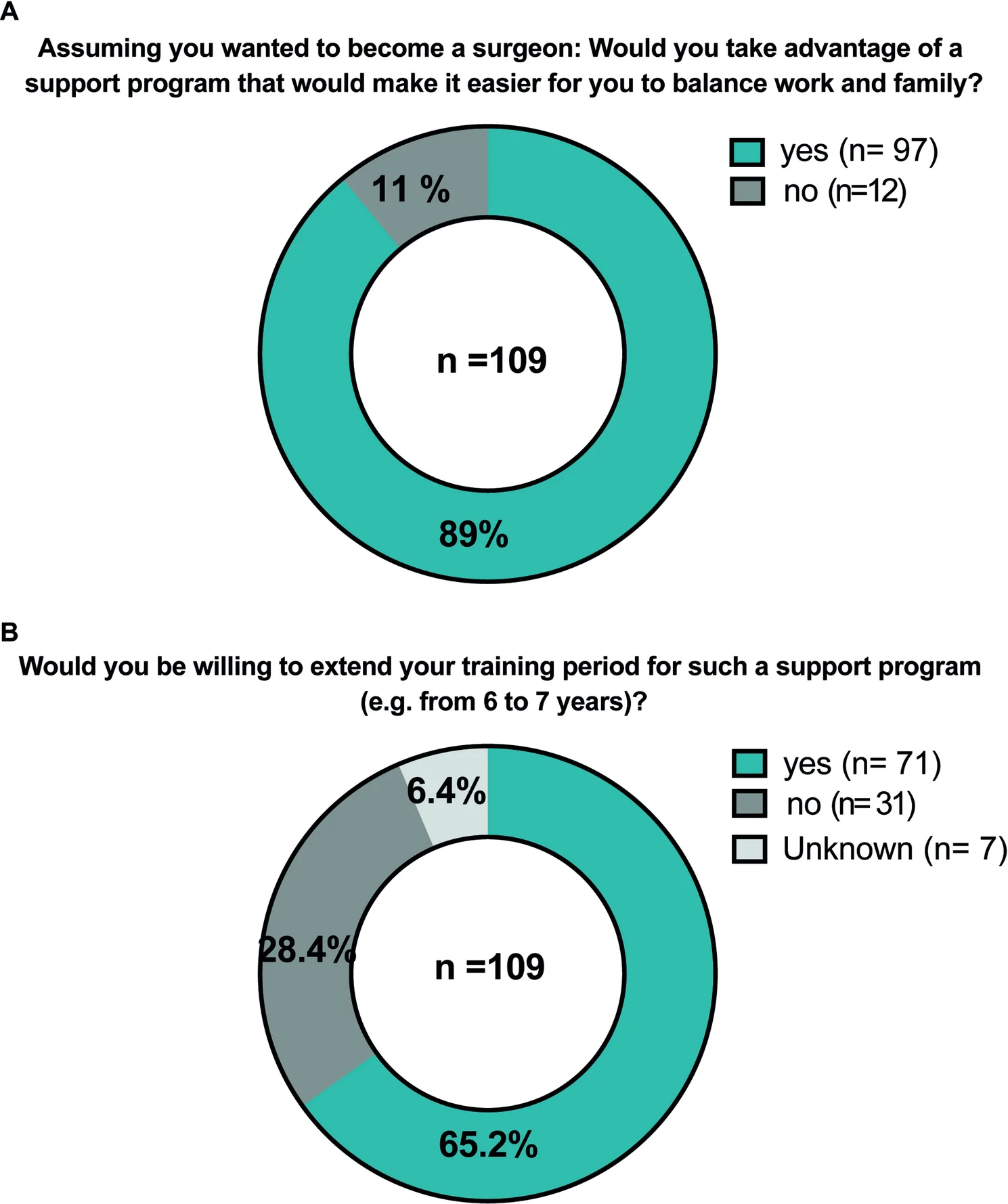
Donut plots of (**A**) Distribution of answers regarding the participants’ willingness to accept support programs aiming to improve work-life balance and (**B**) to accept an extended training period are shown. Percentages, as well as absolute numbers are shown

Finally, we evaluated students’ interest in support programs that facilitate balancing professional and family obligations (Fig. 5). Overall, 89.0% (*n* = 97) of participants expressed interest in such unspecified programs (Fig. 5A), and 65.1% (*n* = 71) would be willing to accept an extension of their surgical residency, while 28.4% would not and 6.4% were undecided (Fig. 5B).

## Discussion

This single-centre exploratory analysis using an investigator-developed survey revealed that, even among medical students who chose an elective in neurosurgery, only one third would pursue a career in surgery (Fig. 2A). In our cohort, this proportion was numerically lower among female than among male students (30.6% vs. 41.7%); however, this difference was not statistically significant and does not allow conclusions about gender-specific career preferences. Previous research has described gender disparities in surgical careers (see below), and this aspect should therefore be investigated in larger, gender-balanced cohorts. Given the growing female majority among medical students and the increasing emphasis on work-life balance [12], it is especially important to pave the way for young talents to pursue a surgical career.

### Perception of the surgical stereotype and lifestyle

Regarding the perceived image of surgeons, most participating students associated surgical careers with a competitive and manually skilled professional who maintains emotional distance from patients, spends comparatively little time interacting with them while they are awake, and is simultaneously dedicated extensively to their career, burdened not only by extended working hours but also by demands on their physical and mental health (Figs. 2 and 3). This is in line with other study results evaluating students’ perception and persisting stereotypes associated with surgical sub-specialties, including neurosurgery [13]. Some of these perceptions may reflect observations of working conditions that have been empirically demonstrated, including extended working hours and the associated physical and emotional burden experienced by surgical trainees [5, 10].

There is evidence, that indeed working hours in surgical sub-specialties exceed the average for other medical subspecialities and especially neurosurgery is known for extended working hours [14–16]. This has impact on the physical and mental well-being and higher rates of burnout in surgical residents compared to their medical counterparts [17, 18]. Nevertheless other perceptions may less likely be supported by empirical evidence and still be influenced by stereotypes, such as the necessity of aptitude for manual dexterity, emotional distance from patients or the limited interaction with awake patients [19]. The prevailing data supports the hypothesis that the image of surgeons held by medical students is often a result of preconceived ideas and stereotypes, often cultivated by media, rather than an accurate reflection of the reality of the profession [11]. This image often resolves once students have gained more insight into the clinical practice of surgery. In this regard, we have to state that our cohort of students were asked to answer questions about surgical professions, however their answer patterns might be influenced by their pre-existing neurosurgical perceptions, although they had not yet completed their neurosurgical course.

Offered possibilities for students in early stages of their medical education are often lacking, leading to a delayed perception of the actual surgical working environment and increases possible misperception and prejudices [13, 20]. Especially as sub-specialties as neurosurgery are not always mandatory parts of medical education, a lack of authentic insights can increase misconceptions and preconceived ideas, in this regard our neurosurgical course might help to refine and clarify the perspective and perception of students at their beginning of medical education.

In addition to media portrayals, students’ perceptions are likely shaped by the attitudes and behaviors of clinical role models encountered during medical training. Importantly, these perceptions may therefore arise not only from stereotypes but also from rational inferences based on observed clinical practice. These observations may provide valuable insights into the surgical profession but are also highly context dependent. Short-term observerships, as our neurosurgical one may capture only selected aspects of everyday clinical practice, while surgeons may naturally present themselves in a particularly professional manner when interacting with students. Likewise, challenges such as uncertainty, emotional burden, or work-related stress may be discussed less openly in these settings. Consequently, students’ perceptions may be influenced not only by stereotypes but also by limited and potentially incomplete clinical impressions.

### Gender disparities in surgery

In our survey, more than half of the participants agreed that surgery is a male-dominated field, and female students in particular agreed with the statement “male residents have an advantage in pursuing a career in surgery” (Fig. 4B). In addition, participants associated surgeons with competitiveness and emotional distance, traits that reflect masculine gender-normative stereotypes [21–23].

The literature reports a lack of female role models as well as a gender-related promotion gap in surgical sub-specialties. Although the number of female residents in surgery increases, female surgeons are less likely to be promoted or even account for leadership positions [24–26]. Additionally, female residents face higher stress levels, are at higher risk for burnout and often experience gender associated microaggression and discrimination by their co-workers and patients [27–29].

These findings may help explain why many medical students perceive surgery as a male-dominated field. Persistent gender disparities in leadership positions, role-models and workplace experiences are likely to shape students’ perceptions of surgical careers. The observed gender differences within our exploratory analysis in our cohort are consistent with previous reports describing a disproportionate psychological burden among female surgical trainees. However, these findings should be interpreted as perceptions that may be influenced by both observed workplace realities and individual perception of stereotypically masculine traits, rather than as a comprehensive representation of the surgical profession [30].

Almost two thirds of the participants agreed that a surgical career is difficult to combine with family planning (Fig. 4C). Agreement was numerically higher among female students, although this difference was not statistically significant. Recent surveys conducted by neuro-oncological, German and European societies of neurosurgery and interdisciplinary neurooncology have reported the relevance of this issue, particularly among female clinicians and thereby support the medical students’ perception with empirical evidence [24, 31, 32].

As self-fulfillment and a focus on work-life balance are rated as more relevant in the current generation of medical students, the availability of childcare programs and opportunities for maternity or paternity leave might have an increasing impact on career choices of generation Y medical students [7]. Especially role models and early insights into surgical career options and the compatibility of a career in surgery and family life might address this problem and help students to gain realistic impressions.

### Need for support programs and role models

In our cohort, 89.0% of participants expressed interest in potential support programs for balancing a surgical career with family life (Fig. 5A). Furthermore, 65.1% of the participants would even accept an extension of their surgical residency training by one year (Fig. 5B). Although the survey referred to an abstract support program without specifying its exact structure or implementation, these findings suggest that many students place considerable value on structural measures facilitating work–family compatibility. This is in line with findings of low-income countries which revealed that availability of day care facilities for children enhances willingness to become a neurosurgeon [33]. The willingness to accept a prolonged training period may further indicate that participants perceive improved support during surgical residency as sufficiently important to outweigh a potential extension of training. The high acceptance of structured support besides clinical training is in line with students in their last year expressing the need for structured teaching leadership, communication and career management skills [34]. The generation currently entering the medical field faces a lack of representation and role models, which may be partly compensated by structured mentoring and support programs [31, 35].

Experience from recently generated structured mentoring programs, especially for young female clinicians show, that there is a great acceptance and need for support [32]. Of note, the literature has emphasized the necessity for change and the implementation of sustainable resident programs, incorporating sufficient time for personal development and private life [36]. Legislative changes, such as working hour restrictions have been shown to have not only a positive impact on physicians’ health but also on increasing patients’ safety [37]. However, a negative effect on surgical training is also evident due to decreased surgical exposure during training [38]. Whether early information about such structural improvements influences medical students’ career choices remains to be investigated in future studies.

### Limitations of the study

The main limitation of this study is the preselection of the study collective. The survey was conducted in preclinical medical students who decided to spend an elective in neurosurgery. Students with a primary interest in non-surgical or more conservative specialties may therefore be underrepresented. Another consideration might be, that the students enrolling for a neurosurgical elective might only represent a specific subgroup of medical students and may differ even from those generally interested in surgical disciplines, as neurosurgery may attract individuals with particularly strong interests and perceptions. To minimize the influence of the elective itself on participants’ perceptions, the survey was administered before the clinical course.

The survey asked for perceptions regarding surgery in general. Nevertheless, the students in our cohort chose to do an elective in neurosurgery before completing the questionnaire. Therefore, their responses may have been influenced by perceptions of neurosurgery in particular and may not fully reflect their views of surgical specialties more broadly. Gender-based differences in agreement and disagreement must be interpreted cautiously because of the unbalanced gender distribution and the small male subgroup within our cohort. Therefore, answer patterns should be re-assessed in larger and more gender-balanced study cohorts. Moreover, dichotomizing Likert responses may lead to loss of information, therefore isolated p values close to 0.05 should be interpreted cautiously.

Overall, the participating students perceived surgical careers as being characterized by persistent gender disparities, demanding working conditions—including long working hours and substantial physical and mental burden—and limited compatibility with family life. However, whether these are the underlying causes why only one third of students aspire to a career in surgery cannot be resolved based on the present data. Most students expressed interest in the abstract idea of a support program. Nevertheless, these findings should be interpreted with caution, as the survey did not define the specific characteristics of such a program, including its impact on operative exposure, remuneration, career progression, or whether participation would be voluntary. Therefore, our results should be viewed as reflecting interest in supportive training concepts rather than as evidence for the effectiveness of any specific workforce intervention.

## Conclusion

Overall, survey participants of our investigator-developed questionnaire in their early stage of medical education tend to rate surgical disciplines as demanding, competitive and difficult to balance with private life. This finding is indicative of a general negative perception of surgical subspecialties most likely including neurosurgery. The results of the study show the interest of students in the implementation of programs that support the reconciliation of professional and family life. However, as this exploratory survey within a preselected study population with uneven gender distribution can only provide descriptive data regarding students’ perceptions and their willingness to consider a potential support program, further studies, especially with concrete evaluation of support programs are needed to investigate implications on career choices or workforce interventions.

## Supplementary Information

Supplementary Material 1.

Supplementary Material 2.

Supplementary Material 3.

Supplementary Material 4.

## Data Availability

All data analyzed during this study are included in this published article and its supplementary information files.
