# Supplementary material for: Pre-clinical medical students’ perception of surgical professions and balance with personal life – a single-centre exploratory analysis
Source: BMC Med Educ. 2026 Nov 13;26:1856. doi: 10.1186/s12909-026-10822-6 (PMC13761189; doi:10.1186/s12909-026-10822-6)
Supplement: Supplementary file 1 [file 12909_2026_10822_MOESM1_ESM.docx]

**Supplementary Material 1**

**Reflection Questions**

1. Briefly describe your opinion on surgeons and the field of surgery before the internship (stereotypes, expectations, character traits, working methods).
2. What do you understand by "professional medical conduct"?
3. What physician roles did you experience during your elective? Reflect on them!
4. How did you experience the doctor-doctor and doctor-patient relationships?
5. Were there differences between individual doctors? What physician roles did you observe?
6. How did you perceive interprofessional collaboration? What was the relationship between doctors and other professional groups, and which physician roles were relevant here?
7. Are there things/situations that you would handle differently if you could?
8. Did the elective change your perception of the "physician role"? Did you notice roles that you had previously been unaware of or had not consciously perceived?
9. Has your view/opinion of the field of surgery or surgeons changed as a result of the elective?

Embed these questions into a brief description of your experienced day and submit your report as a PDF to the organizer.
