# Supplementary material for: Pre-clinical medical students’ perception of surgical professions and balance with personal life – a single-centre exploratory analysis
Source: BMC Med Educ. 2026 Nov 13;26:1856. doi: 10.1186/s12909-026-10822-6 (PMC13761189; doi:10.1186/s12909-026-10822-6)
Supplement: Supplementary file 2 [file 12909_2026_10822_MOESM2_ESM.docx]

**Supplementary Materials 2**

**Survey on the Role Perception of Surgeons**

Dear students,

you have registered for the preclinical elective "Everyday Life in Neurosurgery." As part of this elective, we kindly ask you to participate in this survey on the role image of surgeons. The survey focuses on your personal perception of the profession so far. Participation is voluntary, anonymous, and will take approximately 5 minutes of your time.

Kind regards,

XXX

**1. Your Gender?**

- Female
- Male
- Diverse
- Not disclosed (prefer not to say)

**I aim to pursue further education to become a surgeon after my studies.**

Strongly agree

- Somewhat agree
- Neutral
- Somewhat disagree
- Strongly disagree

**3. I have already had experience in the field of surgery.**

- Strongly agree
- Somewhat agree
- Neutral
- Somewhat disagree
- Strongly disagree

**4. A special manual dexterity is a prerequisite for surgical training.**

- Strongly agree
- Somewhat agree
- Neutral
- Somewhat disagree
- Strongly disagree

**5. The necessary manual skills can be learned by almost anyone during surgical training.**

- Strongly agree
- Somewhat agree
- Neutral
- Somewhat disagree
- Strongly disagree

**6. Surgeons have little contact with conscious patients.**

- Strongly agree
- Somewhat agree
- Neutral
- Somewhat disagree
- Strongly disagree

**7. Surgeons prefer emotional distance from their patients.**

- Strongly agree
- Somewhat agree
- Neutral
- Somewhat disagree
- Strongly disagree

**8. Surgical training is associated with a high level of competition among trainees.**

- Strongly agree
- Somewhat agree
- Neutral
- Somewhat disagree
- Strongly disagree

**9. Surgery is a typically male-dominated field.**

- Strongly agree
- Somewhat agree
- Neutral
- Somewhat disagree
- Strongly disagree

**10. Male surgical trainees have an easier time advancing in their surgical careers.**

- Strongly agree
- Somewhat agree
- Neutral
- Somewhat disagree
- Strongly disagree

**11. The field of surgery is associated with longer working hours compared to other medical fields.**

- Strongly agree
- Somewhat agree
- Neutral
- Somewhat disagree
- Strongly disagree

**12. Thedaily work as a surgeon is physically more demanding than in other medical fields.**

- Strongly agree
- Somewhat agree
- Neutral
- Somewhat disagree
- Strongly disagree

**13. The daily work as a surgeon is mentally more stressful than in other medical fields.**

- Strongly agree
- Somewhat agree
- Neutral
- Somewhat disagree
- Strongly disagree

**14. The surgical profession is more detrimental to health compared to other medical fields.**

- Strongly agree
- Somewhat agree
- Neutral
- Somewhat disagree
- Strongly disagree

**15. The surgical profession is difficult to reconcile with family planning?**

- Strongly agree
- Somewhat agree
- Neutral
- Somewhat disagree
- Strongly disagree

**16. If you were to become a surgeon, would you take advantage of a support program that facilitates work-life balance?**

- Strongly agree
- Somewhat agree
- Neutral
- Somewhat disagree
- Strongly disagree

**17. Would you be willing to extend your training period (e.g., from 6 to 7 years) to participate in such a support program?**

- Yes
- No
- Other (please specify)

**18. Thank you for your participation!**
Do you have any comments or feedback on the survey? You may provide them here.
